# Overall survival prediction in metastatic castration-resistant prostate cancer treated with radium-223

**DOI:** 10.1590/S1677-5538.IBJU.2019.0343

**Published:** 2020-03-12

**Authors:** Monica Vidal, Alejandro Delgado, Carlos Martinez, José Jaime Correa, Isabel Cristina Durango

**Affiliations:** 1 Department of Radiology Hospital Pablo Tobon Uribe MedellinAntioquia Colombia Department of Radiology, Hospital Pablo Tobon Uribe, Medellin, Antioquia, Colombia;; 2 Department of Urology Hospital Pablo Tobon Uribe MedellinAntioquia Colombia Department of Urology, Hospital Pablo Tobon Uribe, Medellin, Antioquia, Colombia;; 3 Department of Oncology Hospital Pablo Tobon Uribe MedellinAntioquia Colombia Department of Oncology, Hospital Pablo Tobon Uribe, Medellin, Antioquia, Colombia

**Keywords:** Radium-223 [Supplementary Concept], Castration, Prostatic Neoplasms

## Abstract

**Objective:**

Radium-223(223Ra) is indicated for patients (p) with metastatic castration resistant prostate cancer (mCRCP).

**Objectives:**

The aim of this study was to evaluate the role of baseline clinical variables associated with overall survival (OS) and toxicity of 223Ra. Its purpose was to identify the factors that can predict a better response to treatment and provide information regarding the most appropriate time for the application of 223Ra.

**Materials and Methods:**

Prospective study in 40p with mCRPC treated with 223Ra. End points were OS, progression-free survival and time to progression. The follow-up parameters were: doses received, hemoglobin (Hb), absolute neutrophil count (ANC), platelet count (PC), prostate specific antigen (PSA), alkaline phosphatase (ALP), Visual Analogue Scale for pain, Eastern Cooperative Oncology Group (ECOG) and WHO’s Cancer Pain Ladder. The use of other treatments was also evaluated.

**Results:**

Median OS was 17.1 months(mo) (CI95%6.5-27.7); 26/40p received complete treatment of 223Ra, without reaching a median OS and 14p received incomplete treatment with a median OS 13.6mo(CI95%1.6-25.6). Median follow-up was 11.2mo (range:1.3-45.2). The univariate analysis showed that factors as VAS, ECOG, Hb and ALP values were independently associated with OS. First line treatment with 223Ra was started in 11/40p, while 19p had been heavily pre-treated and 13p received concomitant treatment.

**Conclusions:**

223Ra therapy require an adequate selection of patients to obtain the greatest clinical benefit. Low basal Hb, hight basal ALP, bone marrow involvement and an altered ECOG were the main factors that decreased OS in our patients. 223Ra should be considered relatively early in the course of treatment.

## INTRODUCTION

The frequency of new cases of prostate cancer reported by The Global Cancer Observatory was 12.712 in Colombia for 2018 ( 1 ). Additionally, the incidence of metastatic castration-resistant prostate cancer (mCRPC) is increasing, of which the most common are bone metastases. Mortality secondary to mCRPC is related to the metastatic event, therefore, the increasing incidence of bone metastases represents an ideal target for improving outcomes. Bone metastases are a common cause of morbidity and mortality and pose a secondary economic burden on healthcare. Skeletal related events (SREs) continue to be a major cause of disability, diminished quality of life (QoL), and increased cost for treatment of complications ( 2 ).

Until 2004, chemotherapy was the main treatment for mCRPC. Since then, different strategies have emerged with novel agents to manipulate the androgen-receptor, targeting the immune system and treating the bone micro-environment.

Traditionally, bone pain has been managed with analgesics, external beam radiation-therapy (EBRT), and beta-emitting radioisotopes. While some of the current standard treatments, such as bisphosphonates, rank ligand inhibitors, and bone-seeking beta-emitters like strontium, have been shown to length the progression time, none have demonstrated a survival advantage ( 3 ). The development of others agents, including cabazitaxel, cellular immunotherapy-(sipuleucel-T), androgen biosynthesis inhibitors-(abiraterone), androgen receptor antagonists-(enzalutamide), and targeted therapy for bone metastases with Radium-223 dichloride (223Ra) ( 4 ) have been of great benefit, but there is uncertainty regarding the optimal use of these treatments in sequence and in combination.

223Ra is the sixth novel agent to be added to the treatment of mCRPC, having been approved by the Food and Drug Administration on May, 2013, and this treatment was later incorporated into the National Comprehensive Cancer Network guidelines ( 5 , 6 ). This approval was based on the results of the randomized, double-blinded, multinational clinical trial titled ALSYMPCA-(ALpharadin in SYMptomatic Prostate CAncer) ( 7 ).

ALSYMPCA compared 223Ra with placebo in men with mCRPC with symptomatic bone metastases and no visceral metastases. In this clinical trial incorporating 307 patients (p) in the placebo arm and 614p in the treatment arm. The clinical trial demonstrated a 3.6 month (mo) overall survival (OS) benefit with 223Ra, in comparison to the placebo (median14.9 vs.11.3mo HR 0.70, 95% CI: 0.58-0.83, p <0.001) ( 7 ).

223Ra is an alpha emitter and calcium-mimetic that targets the hydroxyapatite matrix in the bone, thereby accumulating in areas of active bone remodeling and formation, such as sites of osteoblastic bone metastases ( 8 - 10 ). Despite the increasing clinical use of 223Ra in mCRPC, clinical variables that may predict responses are still difficult to identify ( 11 , 12 ). The aim of this single-center prospective study was to evaluate the role of baseline clinical variables associated with the OS and toxicity of 223Ra therapy. Its purpose was to identify the factors that can predict a better response to treatment and provide information regarding the most appropriate time for the application of 223Ra.

## MATERIALS AND METHODS

### Study design

This prospective study was conducted between November 2014 and April 2018 with 40p with mCRPC treated with 223Ra in the nuclear medicine department at Pablo Tobón Uribe Hospital. The study was approved by the local ethical committee and conducted in accordance with Helsinki Declaration.

### Patients

Patients were included if they had bone pain, two or more bone metastases on bone-scintigraphy, and absence of visceral metastasis in thoracic and abdomino-pelvic-CT. Before the first administration of 223Ra, the absolute neutrophil count (ANC) was ≥1.5x109/L, hemoglobin (Hb) was ≥10g/dL, and the platelet count (PC) was ≥100x109/L. During subsequent administrations, the ANC was ≥1x109/L, and PC was ≥50x109/L.

### 223Ra treatment

223Ra is a emitting alpha-particle with a short range of 2-10 cell bodies (10μm) and a physical half-life of 11.43 days. It has a complicated decay scheme with a series of six daughter products before decaying to stable lead. The total emitted energy is 28.2 MeV, of which 93.5% are α-emissions (average energy of 5.78MeV), less than 3.6% is β-particle, and less than 1.1% are γ-emissions (154keV). This results in a low signal which can present challenges for quantitative imaging, but nevertheless, introduces the potential for individualized biodistribution studies. The treatment consisted of 6 cycles every 4 weeks. The standard dose is 55kBq/kg.

### Evaluation and follow-up

Before and after treatment, the patients were clinically evaluated using Eastern Cooperative Oncology Group (ECOG), WHO’s Cancer Pain Ladder (CPL) and Visual Analogue Scale for pain (VAS) to evaluate the level of functioning, the decrease in consumption of opioid analgesics, and the reduction of skeletal pain, respectively ( Table-1 ). Bone-scintigraphy was achieved before and after 223Ra-treatment. Skeletal tumor burden was classified as low when the number of bone metastases was between 2-6, intermediate >6, and high burden in the presence of diffuse disease (superscan). Laboratory tests were assessed before, during, and after 223Ra treatment with ANC, Hb, PC, prostate specific antigen (PSA), lactate dehydrogenase (LDH), and alkaline phosphatase (ALP). The use of chemotherapy, abiraterone, enzalutamide, bisphosphonates, and EBRT was also evaluated.

**Table 1:** Classification of ECOG, CPL and VAS.

Visual analogue scale for pain (VAS)		WHO´s Cancer Pain Ladder (CPL)	
0	No	0	No pain. Analgesia not required
1	Mild	1	Mild pain. No opioid use
2		2	Moderate pain. Occasional opioid use
3		3	Severe pain. Daily opioid use
		
4	Moderate	**ECOG STATUS**	
	
5		0	Asymptomatic. Fully active, able to carry on all activities without restriction
6		1	Symptomatic. Restricted in physically strenuous activity. Able to carry out work of a light or sedentary nature
7	Severe	2	Symptomatic. <50 % in bed during the day. Ambulatory and cupable of all self-care. Unable to carry out any work activities
8		3	Symptomatic. >50 % in bed. Capable of only limited self-care, confined to bed or chair 50 % or more of waking hours
9		4	Bedbound
10	Worst	5	Death

### Response criteria

Response to treatment was defined as a sustained reduction in skeletal symptoms (VAS), an increase in the level of functioning (ECOG), a decrease in consumption of opioid analgesics (CPL), and a reduction in ALP levels between the first cycle and 1-3mo after the last cycle of 223Ra.

The response rate was defined in terms of ALP change, such as if there was a reduction >25%, if there was a reduction <25%, if there was an increase in ALP <25%, and if there was an increase in ALP levels >25%.

The main endpoint was OS, which was established as from initial 223Ra cycle until either the date of death from any cause or the last follow-up. Patients who were alive at the last follow-up date were censored. Other factors were evaluated, such as progression-free survival (PFS), which was established from initial 223Ra cycle until date of objective tumor progression, death by any cause, or last follow-up, and time to progression (TTP), which was assessed from the date of initial 223Ra cycle to date of objective tumor progression (defined as a lesion progressing in the bone, nodal or visceral lesions). Patients who were alive and did not experience an event (progression or SREs) were censored.

### Evaluation of Toxicity

Safety was assessed on the basis of adverse events, both hematologic, in the clinical laboratory, and gastrointestinal, in physical examination findings. All adverse events that occurred after randomization, within 3mo after the last injection of 223Ra, were reported and evaluated for their potential relationship to the study drug. Only patients with WHO’s grade of 3-4 from the initial cycle of 223Ra to 3mo after the last administered dose were classified as having hematologic toxicity (ht) ( Table-2 ).

**Table 2:** Hematologic toxicity (according to WHO criteria).

	Hb (g/dL)	ANC (/mm3)	Platelets (/mm^3^)
1	>10.0	>1,500	>75,000
2	8.0 to<10.0	1,000 to<1,500	50,000 to<75,000
3	<8.0 (transfusion indicated)	500 to<1,000	25,000 to<50,000
4	Life-threatening; urgent intervention indicated	<500	<25,000

### Statistical analysis

Patient and clinical characteristics were summarized using descriptive statistics.

Median and range or 95% confidence interval for quantitative variables and categorical variables are shown as number (%). Kaplan-Meier estimates were produced for the cumulative incidence of OS, according to number of cycles of 223Ra, ECOG, basal level of Hb, metastatic involvement in bone bone-scintigraphy, and start of 223Ra-therapy. All statistical analyses were performed using SPSS for Windows.

## RESULTS

Patients had a median age of 72years (range=39-88) and received a total of 183 cycles of 223Ra with a median follow-up time of 11mo (range=1.3-41).

**Figure 1:**
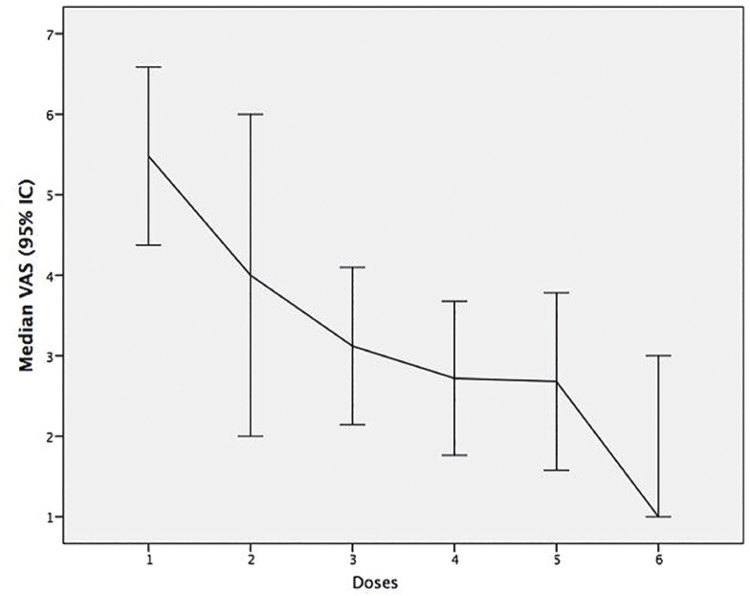
Trend in VAS from the 1st to the 6th223Ra cycles. There is a decrease in pain during complete therapy. The main pain improvement is visualized during the first three cycles of treatment.

25/40p (63%) received the 6 cycles of 223Ra, 1p (2%) received 5 cycles, and 14p (35%) received 4 cycles or less. Out of the 14p who received 4 cycles or less, 1p discontinued the treatment due to unalleviated pain, 1p due to progression, 2p died, 3p for comorbidities, 4p continued the treatment at another hospital and 3p suspended treatment due administrative problems. In Table-3 , the baseline demographic and clinical characteristics of the 40p are described. Of the 26p who finished the treatment, 21p (81%) had a reduction in skeletal pain, the ECOG improved in 9p (35%), and 14p (54%) had a reduction in analgesic requirements ( Figure-1 ).

**Table 3:** Baseline patients’ characteristics.

Baseline variable			n (%) /Value	Range
**Age**		**Mean**	**71**	**39 – 88 years**
ECOG status		Mean	1.92	1-4
		1	8 (20%)	
		2	23 (58%)	
		3	9 (22%)	
CPL		1	11 (27%)	
		2	6 (15%)	
		3	23 (58%)	
VAS		Mild (1-3)	13 (32.5%)	
		Moderade (4-6)	10 (25%)	
		Severe (7-9)	13 (32.5%)	
		Maximum (10)	4 (10%)	
Bone metastases (Bone-Scan)		2 - 6	13 (32%)	
		>6	21 (53%)	
		Superscan	6 (15%)	
Hb		Median	12.8	9.2-16.9
		<13	23 (57.5%)	
		>13	17 (42.5%)	
PSA		Median	93.5	0.26-24784
		0-20	8 (20%)	
		21-99	15 (37%)	
		>100	17 (43%)	
ALP		Median	220	53-2370
		≥200	19 (47%)	
		<200	21 (53%)	
Previous systemic treatments		Abiraterone	16 (40%)	
		Enzalutamide	3 (7%)	
		Chemotherapy	8 (20%)	
		Previous Bone Radiotherapy	19 (47%)	

**Figure 2:**
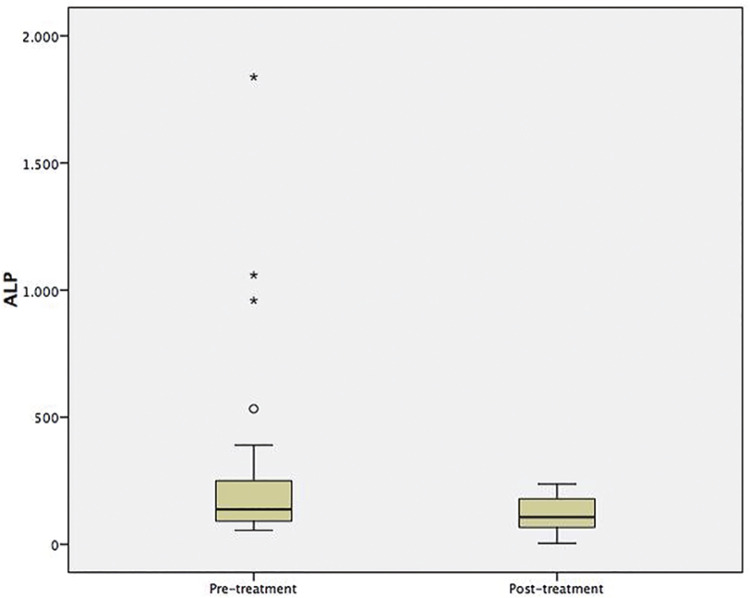
Comparison ALP measurements before and after therapy with 223Ra.

Median value of baseline ALP in our cohort was 210.5U/L- (range=53-2370.8). We found a decrease in serum ALP level in 23/26p (88.5%), 17p with a reduction of >25% and 6p <25%, while 3p had an increase (2p >25% and 1p <25%) ( Figure-2 ). The average reduction was 42.2%- (SD 28.1). Changes in ALP may be a useful marker for monitoring treatment with 223Ra. Further, the ALP baseline level was associated with decrease of OS.

PSA was recorded prior to the first and final cycle and the median PSA showed an upward trend of 93.55ng/mL at the beginning and 142.16ng/mL at the end.

**Figure 3:**
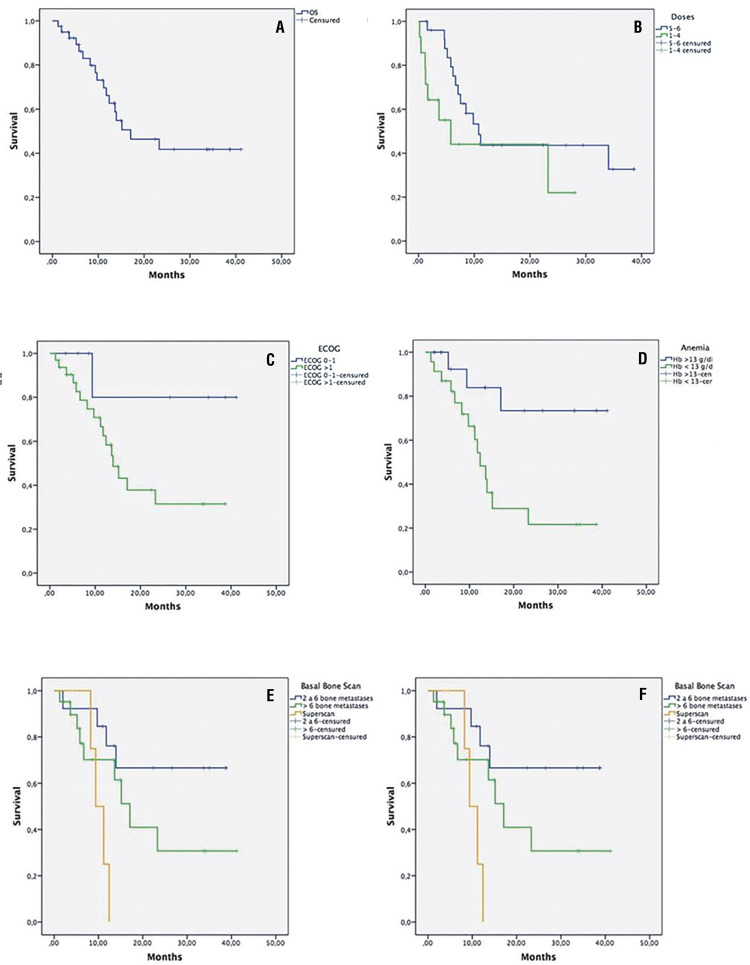
Kaplan-Meier estimate showing the overall survival in our cohort according to different variables. A. Overall survival. B. Number of doses of 223Ra. C. ECOG performance status. D. Basal level Hb. E. Metastatic involvement in bone scan. F. Start of 223Ra therapy.

Median OS was 17.1mo- (range=1.2-41.1, CI 95% 6.5-27.7) ( Figure-3A ), Median follow-up was 11.2mo- (range=1.3-45.2). 26/40p received complete treatments of 223Ra without reaching a median OS, and 14p received incomplete treatments with a median OS of 13.6mo-(CI 95% 1.6-25.6) ( Figure-3B ). PFS was 9.8mo- (CI95%6.6-13). 26p with complete treatments were 11.1mo (CI 95% 8.5-13.8), while those 14p with incomplete treatments were 5mo- (CI95% 2.8-7.3). TTP was 7.1mo- (CI 95% 3.9-10.3).

The univariate analysis showed that the baseline clinical variables, such as ECOG, Hb, bone-scintigraphy, ALP, and PSA, were independently associated with OS ( Figures 3 C-E ). Patients with ECOG >1, baseline Hb levels <13g/dL, superscan on bone-scintigraphy, baseline ALP levels >200U/L, and baseline PSA levels >100ng/mL were associated with an increased risk of death. 11/40p started therapy with 223Ra as the first line without reaching a median OS, while19 had been heavily pre-treated, receiving 223Ra later, with a median OS of 12.4mo, and 13 received treatment concomitant with other therapies (10p: abiraterone and 3p: enzalutamide; 6/13p: bisphosphonates) with a median OS of 13.6mo ( Figure-3F ). 8/40p (20%) were treated with biphosphonates before starting 223Ra, and 15/40p (37%) received concomitant treatment. Results of univariable analysis of OS are shown in Table-4 .

**Table 4:** Univariate analysis of OS in relation to baseline variables.

Variable			Patients (n=40) n (%)	OS	

				Median	CI95%
**ECOG**	0-1		8 (20)	Not reached	
	>1		32 (80)	14	10.2-17.6
**Hb**	>13 g/dL		17 (42)	Not reached	
	<12.9 g/dL		23 (58)	12.4	9.2-15.5
**PSA**	0-20 ng/mL		8 (20)	Not reached	
	21-99 ng/mL		14 (35)	15.2	10.6-19.7
	>100 ng/mL		18 (45)	12.4	7.6-17.2
**ALP**	Missing data		1(2)		
	<200 U/L		19 (48)	Not reached	
	>200 U/L		20 (50)	12.4	7.5-17.3
**Bone-Scan**	2 a 6		13 (32)	Not reached	
	>6		21 (53)	17.1	12.3-21.9
	Superscan		6 (15)	9.4	6.5-12.2
^**223**^ **Ra Therapy**	First line		11 (27)	Not reached	
	Heavily pre-treated before	**Total**	19 (47)	12.4	8.1-16.7
		Abiraterone	10 (25)	9.7	6-13.5
		Enzalutamide	1 (2)		
		Chemotherapy	2 (5)		
		Chemotherapy + abiraterone	4 (10)	15.2	0.1-32.4
		Chemotherapy + enzalutamide + abiraterone	2 (5)		
	Treatment concomitant		13 (32)	13.6	7.8-19.5

Hematologic and gastrointestinal adverse events occurred in 57% and 42% respectively ( Table-5 ). Ht occurred in 7/40p (18%). In 5p it was grade 3 (4p=anemia and 1p=neutropenia) and in 2p it was grade 4 (1p=anemia and neutropenia and 1p=anemia). Of the 7p who developed Ht, 3 received treatments before their 223Ra cycles (1p=chemotherapy and EBRT, 1p=abiraterone and EBRT and 1p=EBRT) and 2p received concurrent abiraterone during 223Ra treatment. 19/40p developed gastrointestinal symptoms, nausea being the most significant in 17p (89%).

**Table 5:** Adverse events graded according to the Common Terminology Criteria for Adverse Events, version 4.0.

p	Doses	Hematologic adverse event (Grades)								Gastrointestinal adverse event (Grades)								

		Anemia		Neutropenia			Thrombocytopenia			Nausea			Vomit			Diarrhea		

		1-2	3-4		1-2	3-4		1-2	3-4		1-2	3-4		1-2	3-4		1-2	3-4
1	4																	
2	2	X			X													
3	2	X			X			X			X			X				
4	6	X																
5	6				X						X						X	
6	6	X				X		X										
7	6				X													
8	6	X																
9	6				X													
10	6	X			X						X			X			X	
11	6		X									X			X			
12	3																	
13	6				X						X			X			X	
14	6				X						X						X	
15	6				X													
16	6	X									X			X				
17	6		X			X					X			X				
18	2	X									X							
19	6				X						X							
20	6																	
21	6	X			X													
22	2	X									X			X				
23	6	X			X			X										
24	3	X																
25	6																	
26	6	X									X							
27	6		X		X			X										
28	2																	
29	2				X													
30	4		X		X			X			X						X	
31	6	X									X							
32	6							X			X							
33	1	X			X													
34	6	X			X			X			X							
35	5				X													
36	6																	
37	4	X															X	
38	3		X		X			X			X			X				
39	6	X			X			X									X	
40	1																	

## DISCUSSION

Over the last years, the treatment for mCRPC has evolved considerably due to the introduction of new therapeutic agents (cabazitaxel/enzalutamide/abiraterone/223Ra). However, the main challenge, finding the best therapeutic sequencing, remains, and it could have a significant impact in terms of clinical improvement and survival. When managing mCRPC patients, many of the bone-related parameters frequently used to determine outcome denote dismal prospects for survival, and so determining which patients will benefit from therapy, in terms of OS, PFS, bone marrow depletion, and SREs, is more difficult ( 13 , 14 ). As a result, there is a need to identify factors that will predict outcome, especially for new therapies, like 223Ra. The aim of this study was to evaluate in a clinical reality the role of baseline clinical variables (ECOG/ALP/Hb/number of bone metastases, and previous treatments) associated with the OS and toxicity of 223Ra-therapy, whose purpose is to identify factors that may predict a better response to treatment and provide information on the most appropriate time for the application of 223Ra. A interdisciplinary approach facilitates identification of patients who are suitable for 223Ra treatment. It has been established that the effectiveness of the treatment on survival is obtained after at least five administrations of 223Ra ( 15 ). For this reason, in this study, complete treatment for patients was defined as 5-6 cycles.

The results of this study are consistent with the findings reported in the ALSYMPCA ( 7 ), confirming that treatment with 223Ra leads to an improvement in pain rate and QoL, which was evaluated with EQ-5D-5L-score and will be reviewed later in a specific publication. Different analyses demonstrated that this treatment is well-tolerated, with a modest objective response rate, and effective in reducing ALP levels, with a clinical benefit and a positive effect on OS ( 16 - 18 ). Our results showed that the response to 223Ra was first clinical and later biochemical, with a moderate decrease in ALP. Taking into consideration some experiences in the literature, we evaluated the variations of ALP to assess the effect of 223Ra treatment ( 19 - 21 ). We observed a significant impact of 223Ra, reducing serum ALP levels by 88.5%, and we noted that the majority of these variations were associated with better pain control, decreased opioid consumption, and better functional status.

In mCRPC patients treated with 223Ra, several baseline prognostic markers associated with OS have been proposed, such as ECOG, ALP and Hb values, and prior systemic treatments. In Table-6 , we describe the baseline clinical characteristics of the patients who received Ra 223 in the three different lines of therapy. Nonetheless, currently, no predictive clinical variable assessing the therapeutic benefit of 223Ra has been identified ( 13 ). The univariate analysis showed that factors like ECOG, VAS, Hb and ALP values were independently associated with OS. Decreased survival rates were seen in patients with basal Hb <13g/dL, superscan on bone-scintigraphy, PSA >100ng/mL, ALP >200U/l, ECOG >1, and those who did not finish the treatment. The use of 223Ra as the first line of therapy showed a greater OS, which suggests that early treatment is beneficial. However, due to the limited number in our sample, we were unable to draw definitive conclusions.

**Table 6:** Patients´ baseline characteristics of treated with 223Ra in the 3 lines of therapy.

^223^Ra therapy		First line (11p)	Concomitant (Abiraterone/Enzalutamide) (13p)	Followed by other treatments (19p)

Baseline variable		n	n	n
Age	Mean	70 years	71 years	71 years
ECOG status	1	3	2	3
	≥2	8	11	16
CPL	1-2	8	2	8
	3	3	11	11
Bone metastases (Bone-Scan)	2-6	6	4	5
	>6	5	7	10
	Superscan	0	2	4
Hb (g/dL)	<12.9	4	10	11
	>13	7	3	8
PSA (ng/mL)	<99.9	10	7	7
	>100	1	6	12
ALP (U/L)	<199.9	6	5	12
	>200	5	8	7

Elba Etchebehere et al. reported a significant benefit in the use of abiraterone concomitant to 223Ra in terms of OS, PFS, and BeFs (univariable: p <0.002 and multivariable: p <0.044). The use of abiraterone with 223Ra reduced the risk of death and SREs by 77% and the risk of progression by 68% ( 22 ). However, a recent analysis from the ERA-223 trial ( 23 ) showed that the simultaneous initiation of the three agents (abiraterone+prednisone/prednisolone with 223Ra) led to an increased risk of fractures and deaths. In our study, in patients who received a concomitance of 223Ra plus abiraterone (10p), OS was lowest, with a median of 13.6mo, without significant changes in the presence of SREs. 16/19p heavily pre-treated had received abiraterone and within this group, the majority had the most unfavorable baseline characteristics, such as Hb <13g/dL, PSA >100ng/mL, ECOG >2, CPL=3, and greater bone compromise, resulting in lower OS ( Table-4 ). Anemia was the most frequent side effect associated with 223Ra ( 24 , 25 ). In the present study, treatment with 223Ra was well-tolerated with only 7.5% of patients experiencing severe anemia (grade 4). The toxicity was manageable and reversible in most cases. The limitations of this study can be attributed to a limited number of patients, heterogeneity of the population and patient’s socio-economic history, considering that some had delays in the administration of the cycles or were changed from hospital. In fact, longer time frames and larger sample sizes are needed to acquire more conclusive results in terms of OS and tolerance to other therapies after treatment with 223Ra. Nevertheless, our study provides valuable information from routine clinical practice in identifying patients who would benefit the most from 223Ra therapy, as well as the most appropriate time to initiate such treatment.

## CONCLUSIONS

223Ra therapy demonstrates maximum efficacy in mCRPC patients who receive the full treatment. It is necessary to select suitable patients who will benefit from this therapy. Basal low Hb levels, bone marrow involvement and an altered performance status were the main factors that decreased survival in our patients. The use of 223Ra as the first line of therapy showed a higher OS, therefore, it should be considered relatively early in the course of treatment. Toxicity was manageable and reversible in most cases.

## ABBREVIATIONS

223Ra: Radium-223
ANC: Absolute neutrophil count
ALP: Alkaline phosphatase
ALSYMPCA: ALpharadin in SYMptomatic Prostate CAncer
BMF: Bone marrow failure
CPL: WHO’s Cancer Pain Ladder
ECOG: Eastern Cooperative Oncology Group
EBRT: External beam radiation-therapy
FDA: Food and Drug Administration
Hb: Hemoglobin
Ht: hematological toxicity
LDH: Lactate dehydrogenase
mCRCP: Metastatic castration resistant prostate cancer
OS: Overall survival
p: Patients
PC: Platelet count
PFS: Progression-free survival
PSA: Prostate specific antigen
SREs: The skeletal related events
TTP: Time to progression
VAS: Visual Analogue Scale for pain
